# Does intensive goose grazing affect breeding waders?

**DOI:** 10.1002/ece3.5923

**Published:** 2019-12-08

**Authors:** Jesper Madsen, Luna Kondrup Marcussen, Niels Knudsen, Thorsten Johannes Skovbjerg Balsby, Kevin Kuhlmann Clausen

**Affiliations:** ^1^ Department of Bioscience Aarhus University Rønde Denmark; ^2^ Naturstregen Esbjerg V Denmark

**Keywords:** barnacle goose, black‐tailed godwit, breeding habitat, lapwing, laser light, meadow birds, shorebirds

## Abstract

Increasing goose population sizes gives rise to conflicts with human socioeconomic interests and in some circumstances conservation interests. Grazing by high abundances of geese in grasslands is postulated to lead to a very short and homogeneous sward height negatively affecting cover for breeding meadow birds and impacting survival of nests and chicks. We studied the effects of spring grazing barnacle geese *Branta leucopsis* and brent geese *Branta bernicla* on occupancy of extensively farmed freshwater grasslands by nesting and brood‐rearing waders on the island Mandø in the Danish Wadden Sea. We hypothesized that goose grazing would lead to a shorter grass sward, negatively affecting the field occupancy by territorial/nesting and chick‐rearing waders, particularly species preferring taller vegetation. Goose grazing led to a short grass sward (<5 cm height) over most of the island. To achieve a variation in sward height, we kept geese off certain fields using laser light. We analyzed effects of field size, sward height, mosaic structure of the vegetation, proximity to shrub as cover for potential predators, and elevation above ground water level as a measure of wetness on field occupancy by nesting and chick‐rearing waders. The analysis indicated that the most important factor explaining field occupancy by nesting redshank *Tringa totanus*, black‐tailed godwit *Limosa limosa*, oystercatcher *Haematopus ostralegu*s and lapwing *Vanellus vanellus* as well as by chick‐rearing black‐tailed godwit and lapwing was short vegetation height. Distance to shrub cover and elevation were less important. Hence, despite very intensive goose grazing, we could not detect any negative effect on the field occupancy by nesting nor chick‐rearing waders, including redshank and black‐tailed godwit, which are known to favor longer vegetation to conceal their nests and hide their chicks. Possible negative effects may be buffered by mosaic structures in fields and proximity to taller vegetation along fences and ditches.

## Graphical abstract

Grazing by high abundances of geese in grasslands is postulated to lead to a very short and homogeneous sward height negatively affecting cover for breeding meadow birds and ultimately impacting survival of nests and chicks. Despite very intensive goose grazing in spring, our field study could not detect any negative effect on the field occupancy by nesting nor chick‐rearing waders.

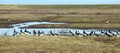

## 1 INTRODUCTION

During recent decades, many wild goose populations in the western Palearctic and North America have increased dramatically due to a combination of protective measures and improved food supplies provided by intensified farming practices in the wintering and staging areas (Ebbinge, 1991; van Eerden, Zijlstra, Roomen, & Timmerman, 1996). The recovery is regarded as a success for nature conservation efforts (Fox & Madsen, 2017), enabling ecosystem and cultural services provided by geese (Buij, Melman, Loonen, & Fox, 2017; Green & Elmberg, 2014). However, the increases have caused socioeconomic conflicts with farming interests due to damage to agricultural crops (Fox, Elmberg, Tombre, & Hessel, 2017) and flight safety (Bradbeer, Rosenquist, Christensen, & Fox, 2017), as well as concerns for impacts on vulnerable ecosystems and biodiversity. Ecosystem impacts may be due to overgrazing of natural habitats (Bakker, Veen, Heerdt, Huig, & Sarneel, 2018; Jano, Jefferies, & Rockwell, 2002; Pedersen, Speed, & Tombre, 2013; Srivastava & Jefferies, 1996) and nutrient input via defecation to aquatic environments used as roost sites (Dessborn, Hessel, & Elmberg, 2016; Jensen et al., 2019). The increasing socioeconomic and ecological conflicts have resulted in calls for the management of populations at national and international levels. Internationally coordinated management plans have been implemented (e.g., Lefebvre et al., 2017; Madsen et al., 2017), or are under implementation under the auspices of the Agreement on the Conservation of African‐Eurasian Migratory Waterbirds (AEWA‐UNEP) (Jensen, Madsen, Nagy, & Lewis, 2018; Powolny et al., 2018).

One of the conservation concerns raised is the potential impact of intensive goose grazing on breeding habitats for meadow birds, many of which are in decline and threatened due to anthropogenic pressures, such as habitat loss on the breeding as well as staging and wintering areas (Pearce‐Higgins et al., 2017). In northwestern Europe, especially the increasing numbers of wintering and spring‐staging barnacle geese *Branta leucopsis* have raised concerns. The Russian‐breeding population, which traditionally wintered in the Wadden Sea area in Northwest Europe, has increased from 20–40,000 in the 1970s to more than 1.2 million in recent years, and the population has started to breed in the temperate Baltic and North Sea region (Jensen et al., 2018). Furthermore, as the population size has grown, the majority of the population has extended its stay in the wintering area until the second half of May, skipping the traditional spring‐staging areas in the Baltic (Eichhorn, Drent, Stahl, Leito, & Alerstam, 2009). The wintering range has expanded to the north and northeast (Jensen et al., 2018).

**Figure 1:**
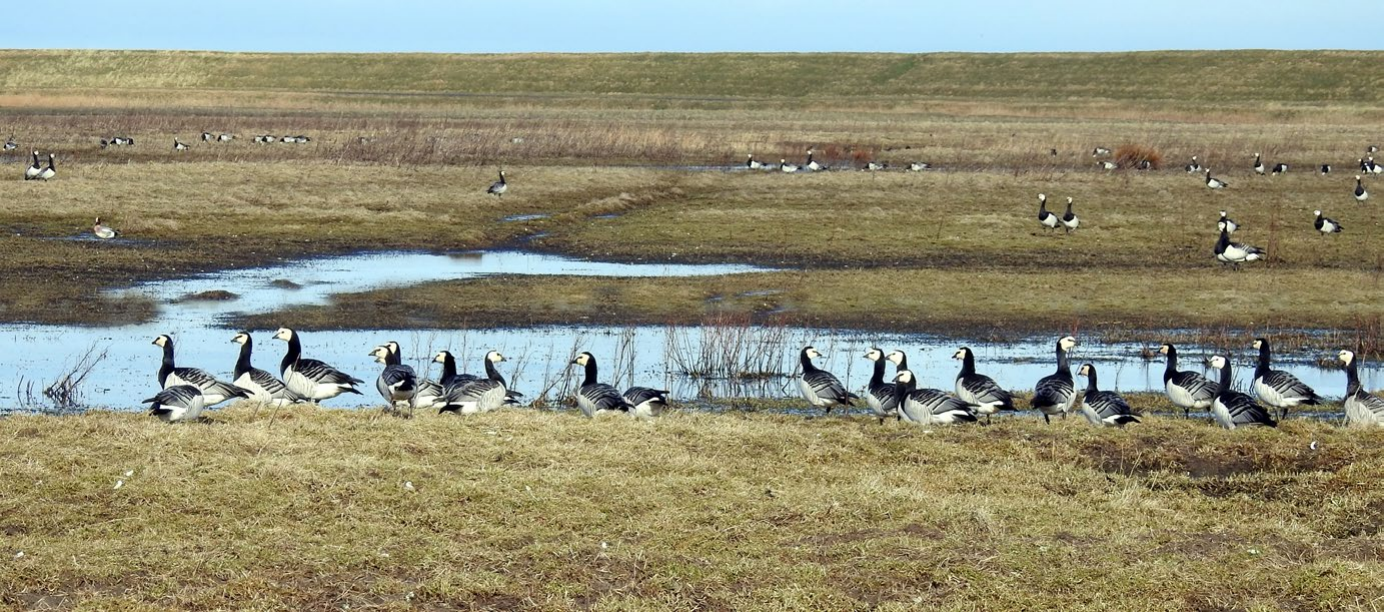
Intensive foraging by flocks of barnacle geese in wet meadows on the island of Mandø results in short swards throughout the spring. In this paper, we examine whether this affects locally breeding waders

Barnacle geese, as well as dark‐bellied brent geese *Branta bernicla bernicla,* predominantly forage on grass in saltmarshes and polder grasslands close to the coast. They congregate in large flocks, often numbering thousands of individuals, and, due to their small bill sizes, the geese are able to bite the grass shoots to a very short level (Durant, Fritz, Blais, & Duncan, 2003). In spring, when grass growth starts, goose flocks frequently revisit fields to feed on the nutritious fresh shoots, and they can maintain the grass sward short (Drent & van der Wal, 1999; van der Graaf, Stahl, & Bakker, 2005)(Figure 1). Saltmarshes and coastal freshwater grasslands are also important breeding and foraging areas for meadow birds, including several species of waders. Some species, such as northern lapwing *Vanellus vanellus* and Eurasian oystercatcher *Haematopus ostralegus*, occupy fields with short vegetation. Here, they place their nests in the vegetation or on bare ground (Milsom et al., 2001). Others, such as common redshank *Tringa totanus* and black‐tailed godwit *Limosa limosa,* prefer slightly longer or more tussocky vegetation where they can conceal their nests (Clausen & Kahlert, 2010; Schekkerman, Teunissen, & Oosterveld, 2008; Smart, Gill, Sutherland, & Watkinson, 2006; Thorup, 2003). Due to the increasing abundances of geese, and their intensive grazing well into the breeding period, there is an increasing concern that geese negatively affect the breeding conditions for some meadow bird species (Jensen et al., 2018). This might be caused by nests becoming more exposed to predation by mammalian and avian predators. Furthermore, recent studies have shown that declines in numbers of waders are largely due to poor chick survival (Roodbergen, Werf, & Hötkerö, 2012) and, potentially, loss of cover due to goose grazing might be a factor leading to a higher risk of chick predation. However, the evidence of a negative impact of goose grazing on breeding meadow birds is poor. Based on a time series analysis of abundances of breeding waders and spring occurrence of barnacle geese in the Netherlands, Kleijn, Winden, Goedhart, and Teunissen (2009) did not find a negative correlation, though localized impacts may have been overlooked.

On the island of Mandø in the Danish Wadden Sea, the local populations of waders have declined during the recent two decades (Laursen & Thorup, 2009). It has been suggested that one of the factors behind this could be a dramatic increase in the numbers of spring‐staging barnacle geese, which have grazed the grass swards short, making the fields unsuitable for nesting and chick‐rearing. Since this concern has also been raised on a wider geographical scale (Jensen et al., 2018), but is poorly documented, there is a need for field studies to provide evidence for possible effects.

We conducted a field study of the effects of grazing by barnacle geese and dark‐bellied brent geese on field occupancy by four species of breeding waders in freshwater polder grasslands on the island of Mandø. We hypothesized that (a) goose grazing would lead to a short grass sward, which would affect the field occupancy by territorial/nesting as well as chick‐rearing waders, and (b) wader species preferring taller vegetation would be most affected. Because the response by waders might be confounded by other factors than geese and sward height, the analysis also incorporated field size, elevation above the ground water level, mosaic structure of the vegetation, and proximity to shrub cover. The shrub vegetation provides shelter and breeding opportunities for red fox *Vulpes vulpes* and crows *Corvus cornix*, which are among the potential predators of breeding waders, their eggs and chicks. A previous study had shown that geese graze the fields very short, almost all over Mandø (Madsen, Knudsen, & Balsby, 2016). In order to achieve a variation in sward height, we experimentally kept geese out of certain parcels of fields by use of laser light. The effectiveness of scaring geese by use of laser is described in Clausen et al. (accepted).

## 2 MATERIAL AND METHODS

### 2.1 Study sites and species

**Figure 2:**
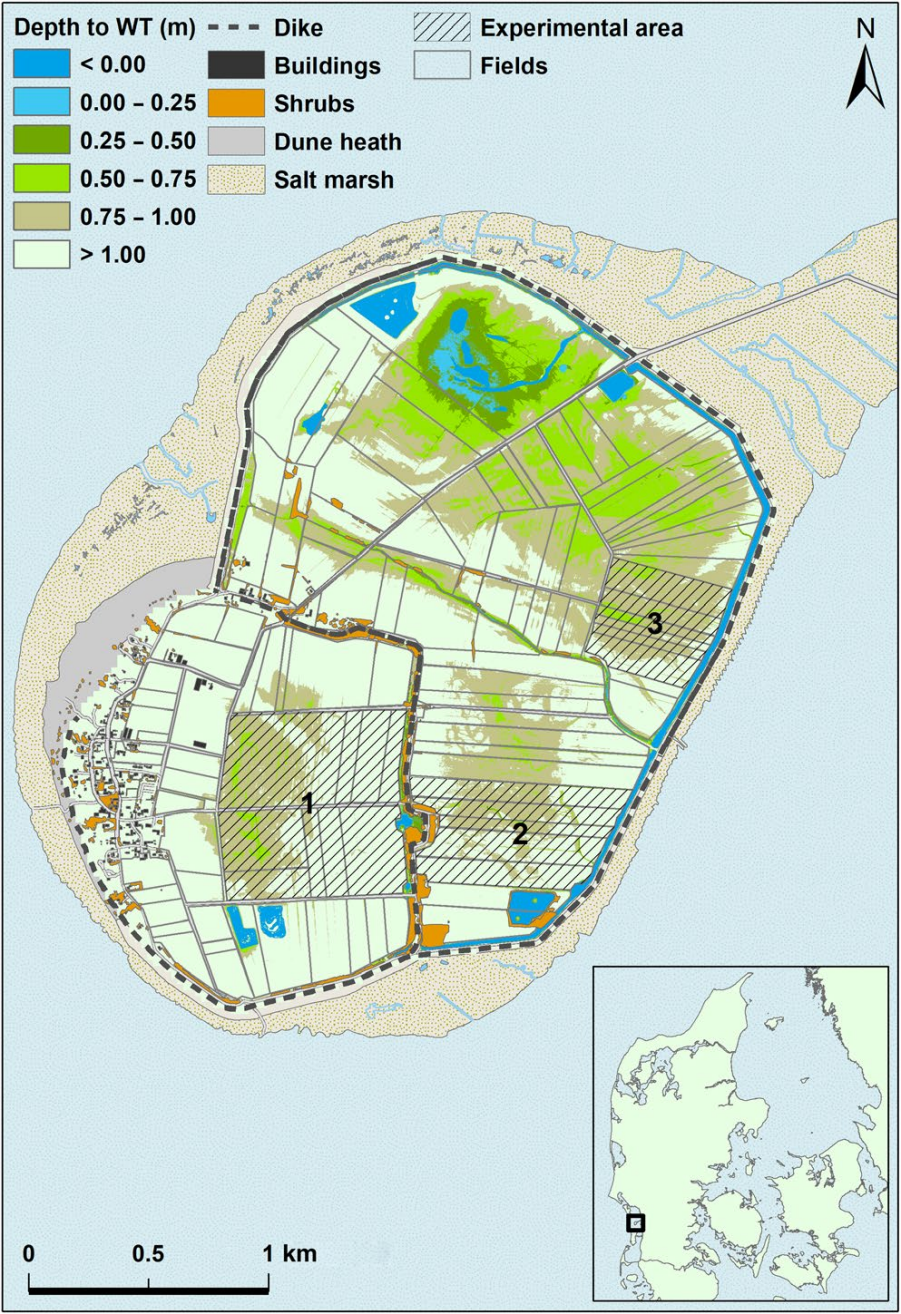
The Mandø study area showing field structure, physical features (buildings, shrub, roads, dikes), elevation of the polders (expressed as distance from water table to terrain elevation) on 1 May 2018, and the three experimental areas where geese were kept out by use of laser light. Insert map shows the position of Mandø in the Danish Wadden Sea

Mandø (lat, long: 55.28, 8.56) is an 8.5 km^2^ island, included in the Danish Wadden Sea NATURA 2000 area and is a designated Wildlife Reserve (Laursen & Thorup, 2009). A dike protects the island from the sea, and an additional dike separates the Mandø village polder from the northern and eastern polders (Figure 2). The island is subject to extensive farming practices, with grasslands used for either sheep or cattle grazing or haycutting; the majority of fields are permanent grasslands, but in some there is a regular reseeding. Only few fields are used for crop rotation with spring cereals. The water levels in the fields are regulated by canals and ditches and sluice gates. Clay pits are found in the interior as well as the outer polders, and shallow‐water areas are found in the northern outer polder (Figure 2). Fields are bordered by ditches and are fenced. Hence, in a narrow zone between fences and ditches, there are narrow areas of higher vegetation. On the inner dike, as well as around some of the larger canals, willow shrubs have formed, and small plantations have been planted in the outer polder and around housing in the inner polder (Figure 2). Mandø is an important breeding area for several species of waders including oystercatcher, redshank, lapwing, and black‐tailed godwit. The latter is a red‐listed breeding bird in Denmark (Laursen & Thorup, 2009).

In March–April, the waders arrive to Mandø to breed in the polders. In 2018, the numbers of breeding waders were estimated at 233 pairs of oystercatcher, 27 pairs of redshank, 137 pairs of lapwing, and 50 pairs of black‐tailed godwit. During spring, Mandø is also an important staging and foraging area for the Russian/NW Europe‐breeding barnacle goose population and the Russian‐breeding dark‐bellied brent goose population. Numbers of geese on Mandø were counted and mapped daily from 15 March to 22 May 2018, where a maximum of *c*. 15,000 barnacle geese and *c*. 3,000 brent geese were recorded. While Mandø has been used by brent geese for several decades, the barnacle geese first started to use the island within the last 15 years, and during the years 2007–2016, peak numbers fluctuated around an average of 17,000 individuals (Madsen et al., 2016); hence, the occurrence in 2018 was typical. On Mandø, both goose species heavily exploit the grasslands as well as the saltmarshes outside the dikes. Barnacle geese leave the island around 15–20 May, while brent geese depart around 25 May.

### 2.2 Surveys of breeding waders (nesting and chick‐rearing phase)

Breeding waders were systematically surveyed and marked on a map with 109 established fields (Figure 2). The surveys resulted in data on occupation by territorial or nesting individuals of all species on a field‐by‐field basis. Observations represent field occupation of the species and do not include information about the timing of nesting, nor survival of nests or chicks. Surveys of breeding black‐tailed godwits, lapwings, oystercatchers, and redshanks were conducted in windows of 2‐ to 3‐day periods, repeated eight times during 25 April to 6 June 2018. The census methods followed a standardized protocol described by Hälterlein et al. (1995). For early breeding species, that is, lapwing and black‐tailed godwit, two surveys conducted during 25 April to 4 May represented the territorial/nesting phase, while subsequent six surveys represented chick‐rearing. In the chick‐rearing phase, numbers of alarming birds and counted broods of black‐tailed godwit and lapwing were noted at 3‐day periods of observations between mid‐May and early June. For oystercatcher and redshank, timing of nesting was prolonged, and we could not clearly discriminate between nesting and chick‐rearing periods during our study period. Because nesting birds were still abundant in early June, we regarded the entire period from April to June as nesting period for these two species. Hence, for the nesting period, we included all four species in the analysis, while for the chick‐rearing period, we only included lapwing and black‐tailed godwit.

### 2.3 Manipulation of goose field use by laser

Three areas were selected for displacement of geese by laser, based on agreements with farmers. Hence, 20 fields of 111 ha in total were chosen (Figure 2). The size and distribution of the experimental areas were designed to be logistically manageable by two laser operators on a daily basis.

Displacement of geese was performed with an Agrilaser Handheld 500^©^, with power <500 mW, wavelength 532 nm (green), and a diameter at aperture of 40–50 mm, which can be used without causing disturbance to breeding waders (Clausen et al., accepted). The displacements took place daily from 26 March to 22 May 2018, where experimental fields were checked throughout the day (with some interruption during transportation and short breaks) from sunrise to sunset by two laser operators. Goose movements were observed from dikes near the experimental areas, and the laser was aimed in front of flocks settling in a given field. Typically, geese quickly took off and flew to other areas of the island. In cases of no or only partial reaction by a goose flock in the experimental areas, the procedure was repeated from a shorter distance until all geese were displaced. Occasionally, few geese occurred in experimental areas out of sight during the day or during the night, but the displacement experiment facilitated areas with a minimum of goose grazing, not completely without.

### 2.4 Hydrology

To investigate potential relationships between field occupation by waders and elevation above the ground water level as a measure of the wetness of fields in the nesting and chick‐rearing periods, we applied a dynamic hydrological MIKE SHE model (Abbott, Bathurst, Cunge, O'Connell, & Rasmussen, 1986) developed by Orbicon. The model of water table depths of Mandø was based on the regional model of Denmark for subarea 4 (Southern Jutland) with a local sampling per 25 × 25 m. MIKE SHE applies dynamic time series of precipitation, potential evaporation, and air temperature (data acquired from the Danish Meteorological Institute). The model was calibrated by seven data loggers (piezometers), which measured water levels on Mandø between 20 June and 10 September 2018 as well as water depths measured in clay pits and shallow‐water areas on 14 September 2018. Furthermore, the model was adjusted to orthophotos from spring 2017 acquired from the Agency of Data Supply and Efficiency (https://sdfe.dk/hent-data/fotos-og-geodanmark-data), which show areas with open water surface.

By subtracting the model depths from a digital terrain model (Danmarks Højdemodel, DHM/Terræn 2016) of Mandø with a sampling per 0.4 × 0.4 m, a map of depth between elevation and water table was achieved. The terrain model was acquired from the Agency of Data Supply and Efficiency (https://www.geodata-info.dk/srv/eng/catalog.search#/metadata/a813e173-b580-459b-87c8-f7407175ef36). Subsequently, the terrain model was combined with depth measurements of lakes and clay pits; hence, the model describes the bottom of waterholes and not the water table for the time of the terrain model measurements. We used the average elevation above the water table on individual fields as an index of field wetness (the lower being the wettest). The index was calculated separately for the nesting period in early May (Figure 2) and chick‐rearing period in early June.

### 2.5 Measurements of goose use and vegetation structure

The field occupation by geese was measured by counts of goose droppings in each field. This gives a reliable description of field use because geese defaecate at short intervals of 3–5 min (Madsen, 1985; Vickery, Sutherland, Watkinson, Lane, & Rowcliffe, 1995). Feces were visible for more than seven weeks on the grassland (tested by 10 fresh feces laid out in a grass field on Mandø in early April 2018). The counts were carried out by use of five randomly sampled 0.54 m^2^ circular plots (radius 0.42 m) per field, and averaged across samples on the same field. The measurements were executed by a team of five people on 2 May (to represent the nesting period) and the last day of laser light displacements, that is, 22 May 2018 (to represent the chick‐rearing period). In the same sampling plots, the vegetation height was measured using a light plastic disk with radius of 6 cm, placed on a stick with a ruler to represent sward height in homogeneous vegetation, that is, not in tussocks which were mostly left ungrazed by geese. Three measurements were randomly made at a distance of up to 1 m around each of the five circular plots. Hence, a total of 15 vegetation height measurements were taken per field and averaged across samples. The occurrence of tussocks (as a potential cover for nests or chicks) was assessed as percentage coverage within each circular plot. Tussocks were defined as patches with taller grass vegetation than the homogeneous grass sward, created by selective grazing by livestock or cattle trampling.

Fields with grazing sheep or cattle were omitted from the sampling, partly because the vegetation heights and tussocks of these fields could be influenced by both grazing livestock and geese, partly because the landowners did not grant permission to fields with lamb. Furthermore, fields which were ploughed or resown were omitted. For the nesting phase, we sampled 87 out of the 109 fields available, while for the chick‐rearing phase, 68 fields.

Distance between shrub vegetation (groups of willow trees or plantations serving as potential hideaway for predators) and the center of each field was derived from the centroid of each field polygon to the nearest shrub using the Near tool in ArcMap 10.4 (ESRI, 2011). Shrub vegetation was digitized from the abovementioned orthophotos from 2017 as well as from the digital terrain model.

### 2.6 Statistical analyses

To analyze the importance of our independent parameters on nesting and chick‐rearing waders, we used generalized linear models. Response parameters included the maximum number of breeding birds counted per field during the nesting phase and the sum of brood days per field across the six counts in the chick‐rearing phase (e.g., if 1, 2, 1, 3, 2, 1 broods were recorded in a given field on the six days, respectively, the number of brood days was 10). We used the sum of observed brood days because the sample size was low and because families are likely to move between different fields that may all satisfy the need for food and protection. These numbers followed an overdispersed poisson distribution, which we corrected for in the model (Littell, Milliken, Stroup, Wolfinger, & Schabenberger, 2006), because some species were not breeding in a large numbers in the surveyed fields. Explanatory fixed parameters included the area of individual fields, proportion of tussocks in the vegetation, average vegetation height, and distance to shrub cover (Table 1). Elevation showed high correlation with several of the other parameters (Table 2). In addition, elevation had less variation than the other parameters, as indicated by the ratio between *SE* and means (Table 1). As elevation is an important factor in habitat choice by breeding waders, we decided to analyze it separately. The other parameters included in the generalized models showed low levels of intercorrelations (Table 2), which enabled reliable parameter estimation in the generalized linear models. Proportion of tussocks and vegetation height were all assessed individually for the nesting and chick‐rearing periods (see methods above), while area and distance to shrub cover were constant between the two periods. To account for effect of field size, we included area in all models tested. We used Proc glimmix in SAS vers 9.4 to analyze the possible model combinations of explanatory parameters without interaction effects for each species.

**Table 1:** Explanatory parameters per field used in the statistical analysis, their mean values, *SE*, and ranges across all plots

Parameter	Definition	Mean ± *SE*	Range
Area	Area (ha) of individual fields	5.10 ± 0.58	0.67–45.70
VegHeight	Average sward height (cm) in 15 plots per field	*n*: 3.90 ± 0.23 *c*: 7.14 ± 0.76	1.07–11.17 0.73–35.47
Tussock	Average proportion (%) in 5 plots per field	*n*: 4.90 ± 0.77 *c*: 2.52 ± 0.53	0–47 0–27
Elevation	Average elevation (m) above ground water level per field	*n*: 1.15 ± 0.04 *c*: 1.24 ± 0.04	0.50–2.50 0.67–2.59
Distance	Distance (m) from field centroid to nearest shrub cover	259 ± 23	24–892

Note

For the dynamic parameters, data are presented for the nesting phase (*n*) and the chick‐rearing phase (*c*), respectively.

**Table 2:** Pearson correlations between parameters in the nesting phase (below the diagonal) and the chick‐rearing phase (above the diagonal)

		Chick‐rearing				
		Area	Tussock	VegHeight	Distance	Elevation
Nesting	Area		0.081	−0.217	0.380	−0.278
	Tussock	−0.010		0.081	−0.063	0.158
	VegHeight	−0.304	0.196		−0.312	0.328
	Distance	0.380	−0.188	−0.450		−0.617
	Elevation	−0.307	0.291	0.569	−0.607	

To figure out which of the parameters primarily affected the number of breeding species we conducted a model selection procedure using the corrected Akaike information criteria (AICc). Rather than a simple null model without any fixed effects, which has no information, we used area as the base model with a simple parameter, as recommended by Burnham, Anderson, and Huyvaert (2011). Hence, the base model assumes that the number of breeding pairs solely depends on the size of the area.

We tested all combinations between area and the four other fixed parameters. To discriminate between these resulting eight models for each species we estimated the delta AICc = AICc_i_‐AICc_min_, calculated the AICc weight and the evidence ratio (ER) for each model for each species (Burnham et al., 2011). A delta AICc smaller than seven is considered to have some support (Burnham et al., 2011). An AICc weight estimates how likely it is that the model is the best model for the given data (Richards, Richards, Whittingham, & Stephens, 2011). The evidence ratio estimates how much more likely a model is compared to the base model (Burnham et al., 2011; Richards et al., 2011).

To illustrate the effect of individual parameters, we used full model averaging from the eight models for each species (Burnham & Anderson, 2002; Symmonds & Moussalli, 2011). We estimated the variance for the full model (Symmonds & Moussalli, 2011) and used this variance to estimate confidence limits for the averaged estimates, although this might be slightly flawed (Turek & Fletcher, 2012).

We analyzed elevation separately due to collinearity with other parameters. The model only had elevation and area as fixed effects and the same random effect as the other models.

We used Pearson correlations to describe the relation between parameters using proc corr in SAS. The number of breeding pairs did not show spatial autocorrelation according to Moran's I for any of the four species (Appendix S1). We used Proc variogram to calculate Moran's I.

## 3 RESULTS

### 3.1 Effect of goose grazing and use of laser to deter geese on vegetation height

**Figure 3:**
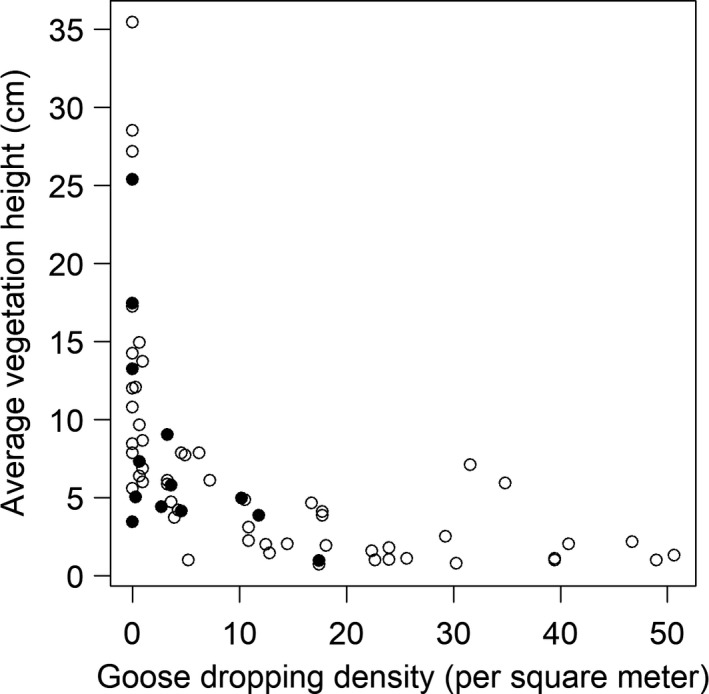
Relationship between average goose dropping density (per m^2^) and average vegetation height (cm) on individual fields on Mandø, measured 22 May 2018. Black points indicate fields in the experimental areas where geese were displaced with lasers

Vegetation height and number of goose droppings on individual fields were inversely correlated in both the nesting phase (Spearman's *ρ* = −0.78, *p* < .001) and chick‐rearing phase (Spearman's *ρ* = −0.81, *p* < .001) (Figure 3). On 22 May 2018, the vegetation canopy was on average 3.3 cm higher in the three experimental areas with displacement of geese by laser than in fields grazed by geese (for further details, see Clausen et al., accepted). Hence, vegetation height reflected goose grazing pressure in the fields.

### 3.2 Factors affecting wader presence

**Figure 4:**
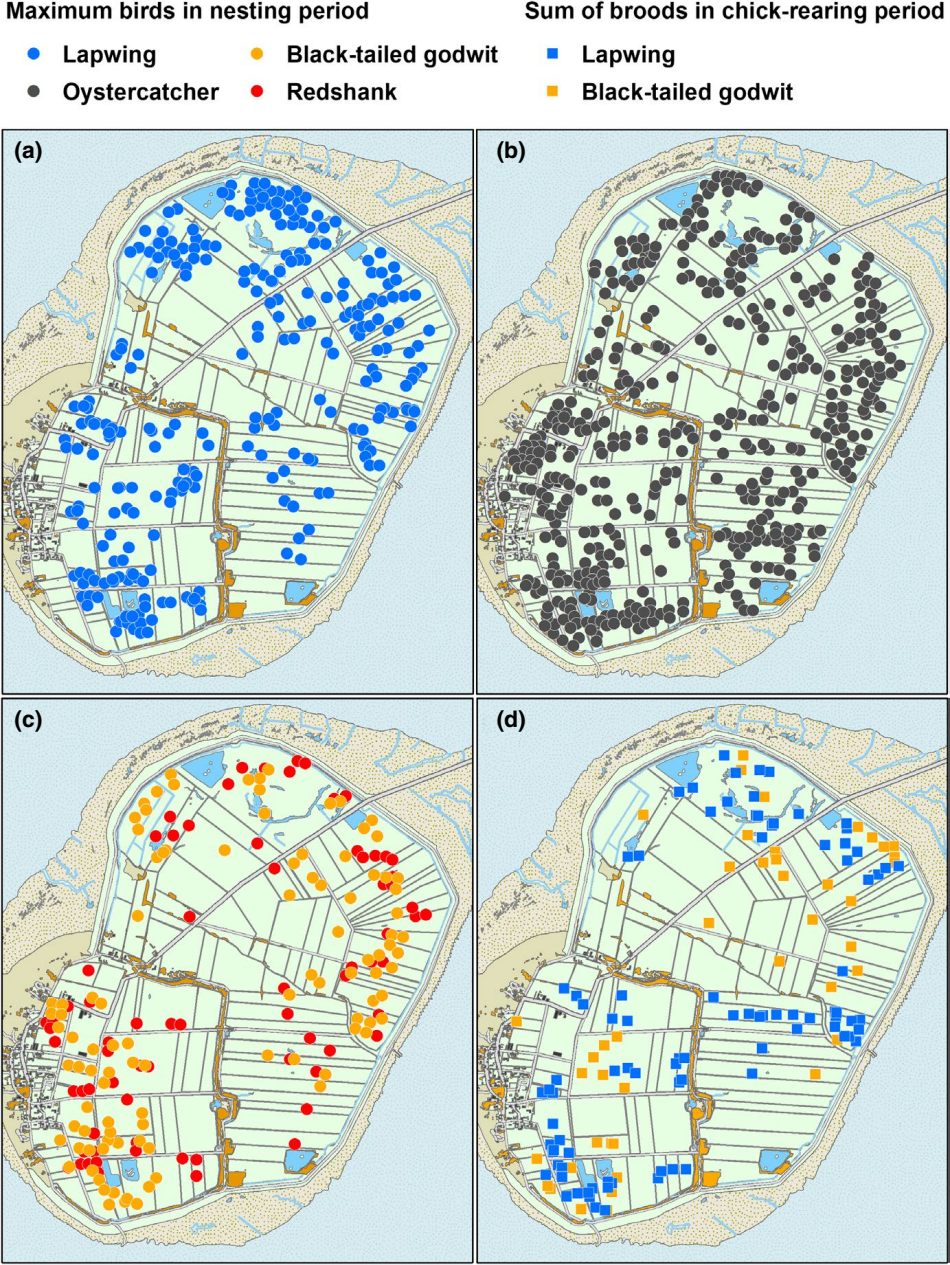
The occupancy of fields on Mandø by the four species of waders during the nesting period (a–c) and for lapwing and black‐tailed godwit during the chick‐rearing period (d). For the nesting phase, the occupancy is expressed by the maximum number of individuals observed per field (across two surveys for lapwing and black‐tailed godwit and eight surveys for redshank and oystercatcher). For the chick‐rearing phase, the cumulative number of records of alarming or brood‐rearing pairs per field is shown (across six surveys)

The spatial distribution of the four species of waders during nesting is shown in Figure 4a–c and the distribution of lapwing and black‐tailed godwit during chick‐rearing in Figure 4d. Below follows a summary of the models that gained most support in describing the environmental factors affecting field occupancy of nesting and chick‐rearing waders. For a full overview of the models, see Appendix S2 (Tables S2.1–S2.6). For all species, it is notable that the model that included all parameters were often not among the best models.

#### 3.2.1 Redshank nesting

Out of eight models, seven had delta AICc values below 7, so many of the parameters had some influence of the number of nesting redshanks. However, two models had higher evidence ratios than the remaining models. Both models included VegHeight as a parameter and one of these models included Distance (Table S2.1). The model with Distance gave the third highest AICc. These results suggest that VegHeight was the most important parameter followed by Distance. Tussock appeared to have little effect on number of nesting redshanks as judged by their effect on delta AICc and evidence ratios.

#### 3.2.2 Godwit nesting

Seven out of eight models had delta AICc values that indicated that they had some support (Table S2.2). Two models, however, had substantially higher evidence ratios, which both included Distance and one model included VegHeight (Table S2.2), which suggests that Distance had more influence than VegHeight. Tussock appeared to have little influence on the number of godwits nesting.

#### 3.2.3 Godwit chick‐rearing

VegHeight was part of the four models with delta AICc < 7. Two models with delta < 7 also included Distance (Table S2.3), whereas Tussock only had little influence on the number of chick‐rearing godwits. VegHeight thus appeared more important than Distance during chick‐rearing.

#### 3.2.4 Oystercatcher nesting

The four models with delta AIC < 7 all included VegHeight (Table S2.4). Two of these models had AICc weights between 32% and 40%. Tussock and Distance did not improve AICc weights and evidence ratios (Table S2.4). VegHeight was thus the only parameter affecting number of nesting oystercatchers.

#### 3.2.5 Lapwing nesting

Two models had delta AICc < 7. Evidence ratios for the models suggested that VegHeight and Distance contributed most to explaining the number of nesting lapwings, and inclusion of Tussock increased the AICc weights from 8.4% to 90.0% (Table S2.5). So, all three parameters appear important for the number of nesting lapwings.

#### 3.2.6 Lapwing chick‐rearing

Two of the eight models had delta AICc < 7 with AICc weights of 26.1%–72.1%. Both models included VegHeight and Tussock (Table S2.6). This suggests that VegHeight and Tussock both were important parameters determining the number of chick‐rearing lapwings, whereas Distance had little influence.

#### 3.2.7 Model‐averaged parameter estimates

The model‐averaged estimates showed similar trends for all species. Generally, the number of nesting birds of all species as well as black‐tailed godwit and lapwing during chick‐rearing showed a negative relation with VegHeight indicating that more individuals used areas with low vegetation height, and positive relations with Area, Distance, and Tussock, documenting that areas with more tussocks and longer distances to shrub were used more (Table 3). It should be noted that Distance and Tussock only were estimated for some of the species in the best models.

**Table 3:** Averaged model parameter estimates including 95% confidence limits and *SE* for four species of waders in the nesting and chick‐rearing phase

		Mean	Lclm	Uclm	*SE*
Nesting					
Lapwing	Area	0.04718	−0.16613	0.26050	0.10884
Lapwing	Tussock	0.02468	−0.20510	0.25446	0.11723
Lapwing	VegHeight	**−0.15809**	−0.52409	0.20790	0.18673
Lapwing	Distance	**0.00119**	−0.10379	0.10617	0.05356
Redshank	Area	0.04870	−0.18113	0.27852	0.11726
Redshank	Tussock	−0.00003	−0.13618	0.13612	0.06947
Redshank	VegHeight	**−0.11694**	−0.45134	0.21746	0.17061
Redshank	Distance	*0.00071*	−0.08591	0.08732	0.04419
Godwit	Area	0.03683	−0.20055	0.27421	0.12111
Godwit	Tussock	0.00612	−0.15052	0.16276	0.07992
Godwit	VegHeight	*−0.05306*	−0.34099	0.23487	0.14690
Godwit	Distance	**0.00103**	−0.09879	0.10086	0.05093
Oystercatcher	Area	0.05371	−0.14300	0.25042	0.10036
Oystercatcher	Tussock	−0.00125	−0.12016	0.11766	0.06067
Oystercatcher	VegHeight	**−0.15558**	−0.48436	0.17319	0.16774
Oystercatcher	Distance	−0.00018	−0.06570	0.06533	0.03343
Chick‐rearing					
Lapwing	Area	0.00001	−0.02412	0.02413	0.01231
Lapwing	Tussock	*0.06247*	−0.22738	0.35232	0.14788
Lapwing	VegHeight	**−0.04651**	−0.37054	0.27752	0.16532
Lapwing	Distance	0.00009	−0.06495	0.06513	0.03318
Godwit	Area	0.00000	−0.02555	0.02556	0.01304
Godwit	Tussock	0.01856	−0.18636	0.22348	0.10455
Godwit	VegHeight	**−0.02294**	−0.33299	0.28711	0.15819
Godwit	Distance	*0.00145*	−0.10950	0.11240	0.05661

Note

The most and second most important parameters for each species are marked with bold and italics, respectively. For nesting lapwing, it was impossible to discriminate between the two most important parameters and both were marked with bold. For oystercatcher, only VegHeight was important.

#### 3.2.8 Elevation

All species showed preference for moist fields as indicated by the negative relation between elevation and the number of nesting pairs for all species and the number chick‐rearing lapwing and black‐tailed godwit (Table 4). Comparison of AICc values for the model with Elevation and the other models (Tables S2.1–S2.6) indicated that Elevation resulted in an inferior fit to the data for most of the models, except for redshank and black‐tailed godwit in the nesting period where the difference in AICc relative to the best model was <7, which lends some support to the model with Elevation.

**Table 4:** Akaike information criterias (AICCs) parameter estimates and *SE* from the generalized linear mixed model with elevation and area for each species in the nesting and chick‐rearing phase

	AICC	Area		Elevation	
		Estimate	*SE*	Estimate	*SE*
Nesting					
Lapwing	392.4	0.060270	0.008869	−0.917300	0.426700
Redshank	198.6	0.059290	0.012040	−0.479900	0.500800
Godwit	248.2	0.044470	0.013060	−0.732800	0.500600
Oystercatcher	418.8	0.058510	0.006473	−0.183700	0.239900
Chick‐rearing					
Lapwing	264.7	0.000007	0.000002	−0.045270	0.644500
Godwit	171.6	0.000005	0.000002	−1.162100	0.784600

## 4 DISCUSSION

Barnacle geese and dark‐bellied brent geese exert a heavy grazing pressure on the polder grasslands of Mandø in spring. Clearly, geese contributed to a low sward on the island, maintained until the second half of May (in most fields <5 cm, except for fields close to the village and farms). Because of high degree of intercorrelation between elevation as a measure of wetness, vegetation height and distance to shrub, we split the analyses. The general pattern from the analyses suggests that the waders had preference for short vegetation and long distance to shrub which outweighed their preference for fields with low elevation. For nesting redshank and godwit, the differences in AICc indicated that elevation could have some support, though other models without elevation gave better fits. These results were expected for oystercatcher and lapwing, which nest in open grassland or on bare ground, but contrary to expectations for redshank and black‐tailed godwit, which conceal their nests in the vegetation. Other studies have found that redshank prefers nesting habitats with a grass sward higher than 5 cm (Smart et al., 2006) and fields with a heterogeneous grassland typology (Verhulst, Kleijn, Loonen, Berendse, & Smit, 2011; Żmihorski et al., 2018).

The experimental use of laser to displace geese had a positive effect on the vegetation height (Clausen et al., accepted), but irrespectively of this potential improvement of sward height, which might have had a positive effect of the numbers of redshank and black‐tailed godwit, short vegetation height turned out to be important for all four nesting species of waders investigated.

For chick‐rearing black‐tailed godwit and lapwing, low vegetation height was also the most important parameter. In particular with regard to black‐tailed godwit, this was unexpected, because it is known from other studies that chicks prefer to stay in relatively tall grass (>15 cm)(Schekkerman & Beintema, 2007) and that shorter vegetation height has negative implications for chick survival (Schekkerman et al., 2008). We did not measure the survival of nests or broods, and it cannot be excluded that low vegetation height may entail an increased risk of predation. A negative effect may be buffered by structural heterogeneity due to tussocks in the fields and taller vegetation along fences and canals providing cover. On Mandø, many fields are long and with a width of <100 m, which means that there is often cover nearby for nests and chicks. On the other hand, these linear habitats might be subject to a high predation risk caused by mammalian predators.

Goose grazing of the sward may also have a positive effect for waders, such as lapwing and redshank which prefer to forage in grasslands with a height < 15 cm (Ausden, Sutherland, & James, 2001). Furthermore, because the barnacle geese and brent geese stay on the island until around 20 and 25 May, respectively, swards are kept low for an extended period, which may be beneficial for late breeding or renesting waders.

Distance to shrub cover was important for several species, in line with other studies which have found that waders prefer wide‐open landscapes (Clausen & Kahlert, 2010; Żmihorski et al., 2018) and that proximity to trees can incur an increased predation risk (Berg, Lindberg, & Källebrink, 1992).

High water tables are known to be important for meadow nesting and chick‐rearing waders, which are dependent on foraging on soil macroinvertebrates (Ausden et al., 2001; Groen et al., 2012; Schekkerman & Beintema, 2007). On Mandø, wetness of fields was also important for the four species of waders, although it did not seem to be the major driver of their field preference. Waders can feed in drills within fields, in low wet patches, and along shallow ponds with grazed margins. Furthermore, on Mandø all fields are relatively close to wet areas. However, except for the northeast corner of the island, the fields are drained by ditches resulting in a low water table, and particularly in the center of the island, many fields dry out in the course of early summer.

## 5 CONCLUSIONS

Barnacle geese and brent geese intensively grazed the swards to a very short height, but we were not able to see any negative effect of the intensive grazing on the field occupancy by nesting nor chick‐rearing waders. On the contrary, short‐grazed fields were preferred by all species, including species which are known to prefer longer vegetation. Possible negative effects of short vegetation may have been buffered by mosaic structures in the fields as well as close proximity to taller vegetation along fences and ditches.

The intensive goose grazing, which is maintained toward the end of May, means that farmers have to delay the release of livestock onto fields and the first mowing of grass by up to one month (N. C. Nielsen pers. comm.). Due to the delays, trampling of nests and mortality of eggs and chicks associated with mowing can be reduced (Kentie, Booth, Hooijmeijer, & Piersma, 2015; Pakanen, Aiko, Luukkonen, & Koivula, 2016). For the farmers, however, these delays have economic consequences, and it is questionable whether current farming practices remain viable, unless a common grassland management scheme is set up for the island. In order to mitigate possible negative effects of goose grazing, such a scheme should also integrate removal of shrub cover to reduce predation risk and raising water tables to improve wader foraging opportunities.

We studied the field occupancy by waders in relation to goose grazing. However, predation rates of nests and chicks over the entire breeding season have not been examined, and hence, it cannot be evaluated if the local breeding wader populations remain self‐supporting. We propose that additional detailed studies are carried out to discern the effects of intensive goose grazing on wader nest and chick survival in various field types with regard to sward height and heterogeneity.

## CONFLICT OF INTEREST

None declared.

## AUTHOR CONTRIBUTIONS

JM and KKC conceived the ideas and designed the methodology; NK, JM, KKC, and LKM collected the data; KKC, TJSB, LKM, and JM analyzed the data; JM, KKC, TJSB, and LKM led the writing of the manuscript. All authors contributed critically to the drafts and gave final approval for publication.

## Data Availability

All data are stored and made publicly available in a database created and maintained by Department of Bioscience, Aarhus University (https://projects.au.dk/da/can/mandoe-monitering/).
