# Supplementary material for: Does intensive goose grazing affect breeding waders?
Source: Ecol Evol. 2019 Dec 8;9(24):14512–22. doi: 10.1002/ece3.5923 (PMC6953592; doi:10.1002/ece3.5923)
Supplement: Supplementary file 1 [file ECE3-9-14512-s001.docx]

Appendix S1

Table S1. Moran’s I test of spatial autocorrelation of the number of breeding pairs for the four studied species.

|  | Observed | Expected | Standard deviation | z | p |
| --- | --- | --- | --- | --- | --- |
| Redshank | -0.0115 | -0.0115 | 0.000116 | -0.013 | 0.9876 |
| Godwit | -0.0114 | -0.0115 | 0.00012 | 0.896 | 0.3704 |
| Oystercatcher | -0.0115 | -0.0115 | 0.00012 | -0.371 | 0.7107 |
| Lapwing | -0.0114 | -0.0115 | 0.00012 | 0.976 | 0.3291 |

Appendix S2. AIC information for models to test the effect of environmental parameters on nesting and chick-rearing waders. ER is relative to base model (Area). For definition of environmental explanatory parameters, see Table 1. The best model is shown in bold and the second best in italics.

Table S2.1. Number of nesting redshank.

| Model parameter | | | | AICc | dAICc | AICc weight | ER |
| --- | --- | --- | --- | --- | --- | --- | --- |
| Area | Tussock | VegHeight | Distance | 194.83 | 2.25 | 8.98% | 4.22 |
| Area | Tussock | VegHeight |  | 194.77 | 2.19 | 9.26% | 4.35 |
| Area | Tussock |  | Distance | 195.66 | 3.08 | 5.93% | 2.79 |
| *Area* |  | *VegHeight* | *Distance* | *192.6* | *0.02* | *27.39%* | *12.87* |
| Area | Tussock |  |  | 199.62 | 7.04 | 0.82% | 0.38 |
| **Area** |  | **VegHeight** |  | **192.58** | **0** | **27.67%** | **13.00** |
| Area |  |  | Distance | 193.46 | 0.88 | 17.82% | 8.37 |
| Area |  |  |  | 197.71 | 5.13 | 2.13% | 1.00 |

Table S2.2. Number of nesting black-tailed godwit.

| Model parameter | | | | AICc | dAICc | AICc weight | ER |
| --- | --- | --- | --- | --- | --- | --- | --- |
| Area | Tussock | VegHeight | Distance | 245.20 | 0.95 | 16.01% | 10.5 |
| Area | Tussock | VegHeight |  | 247.40 | 3.15 | 5.33% | 3.5 |
| Area | Tussock |  | Distance | 245.39 | 1.14 | 14.56% | 9.5 |
| **Area** |  | **VegHeight** | **Distance** | **244.25** | **0.00** | **25.75%** | **16.9** |
| Area | Tussock |  |  | 251.82 | 7.57 | 0.58% | 0.4 |
| Area |  | VegHeight |  | 245.93 | 1.68 | 11.12% | 7.3 |
| *Area* |  |  | *Distance* | *244.30* | *0.05* | *25.11%* | *16.4* |
| Area |  |  |  | 249.90 | 5.65 | 1.53% | 1.0 |

Table S2.3. Number of chick-rearing black-tailed godwit.

| Model parameters | | | | AICc | dAICc | AICc weight | ER |
| --- | --- | --- | --- | --- | --- | --- | --- |
| *Area* | *Tussock* | *VegHeight* | *Distance* | *150.43* | *0.2* | *39.43%* | *1.2x10^5^* |
| Area | Tussock | VegHeight |  | 153.96 | 3.8 | 6.75% | 20952 |
| Area | Tussock |  | Distance | 166.88 | 16.7 | 0.01% | 33 |
| **Area** |  | **VegHeight** | **Distance** | **150.19** | **0.0** | **44.46%** | **1.4x10^5^** |
| Area | Tussock |  |  | 173.21 | 23.0 | <0.001% | 1 |
| Area |  | VegHeight |  | 153.31 | 3.1 | 9.34% | 2.9x10^4^ |
| Area |  |  | Distance | 168.37 | 18.2 | 0.01% | 16 |
| Area |  |  |  | 173.86 | 23.7 | <0.001% | 1 |

Table S2.4. Number of nesting oystercatcher.

| Model parameters | | | | AICc | dAICc | AICc weight | ER |
| --- | --- | --- | --- | --- | --- | --- | --- |
| Area | Tussock | VegHeight | Distance | 396.73 | 2.33 | 12.60% | 3.7x10^4^ |
| Area | Tussock | VegHeight |  | 396.41 | 2.01 | 14.78% | 4.3x10^4^ |
| Area | Tussock |  | Distance | 419.89 | 25.49 | <0.001% | 0.3 |
| *Area* |  | *VegHeight* | *Distance* | *394.85* | *0.45* | *32.24%* | *9.3x10^4^* |
| Area | Tussock |  |  | 418.49 | 24.09 | <0.001% | 0.7 |
| **Area** |  | **VegHeight** |  | **394.4** | **0** | **40.38%** | **1.2x10^5^** |
| Area |  |  | Distance | 418.69 | 24.29 | <0.001% | 0.6 |
| Area |  |  |  | 417.74 | 23.34 | <0.001% | 1 |

Table S2.5. Number of nesting lapwing.

| Model parameters | | | | AICc | dAICc | AICc weight | ER |
| --- | --- | --- | --- | --- | --- | --- | --- |
| **Area** | **Tussock** | **VegHeight** | **Distance** | **363.67** | **0** | **89.98%** | **6.1x10^8^** |
| Area | Tussock | VegHeight |  | 372.56 | 8.89 | 1.06% | 7.1x10^6^ |
| Area | Tussock |  | Distance | 375.56 | 11.89 | 0.24% | 1.6x10^6^ |
| *Area* |  | *VegHeight* | *Distance* | *368.41* | *4.74* | *8.41%* | *5.7x10^7^* |
| Area | Tussock |  |  | 404.79 | 41.12 | <0.001% | 0.712 |
| Area |  | VegHeight |  | 375.27 | 11.6 | 0.27% | 1.8x10^6^ |
| Area |  |  | Distance | 378.72 | 15.05 | 0.05% | 3.3x10^5^ |
| Area |  |  |  | 404.11 | 40.44 | <0.001% | 1 |

Table S2.6. Number of chick-rearing lapwing.

| Model parameter | | | | AICc | dAICc | AICc weight | ER |
| --- | --- | --- | --- | --- | --- | --- | --- |
| *Area* | *Tussock* | *VegHeight* | *Distance* | *214.98* | *2.03* | *26.14%* | *2.2x10^10^* |
| **Area** | **Tussock** | **VegHeight** |  | **212.95** | **0.00** | **72.14%** | **6,1x10^10^** |
| Area | Tussock |  | Distance | 254.32 | 41.37 | <0.001% | 62.80 |
| Area |  | VegHeight | Distance | 223.19 | 10.24 | 0.43% | 3.6x10^8^ |
| Area | Tussock |  |  | 253.52 | 40.57 | <0.001% | 93.69 |
| Area |  | VegHeight |  | 221.00 | 8.05 | 1.29% | 1.1x10^8^ |
| Area |  |  | Distance | 264.00 | 51.05 | <0.001% | 0.50 |
| Area |  |  |  | 262.60 | 49.65 | <0.001% | 1.00 |
